# Atypical Lipomatous Tumours vs. Lipomas: A Multimodal Diagnostic Approach

**DOI:** 10.3390/diagnostics15121538

**Published:** 2025-06-17

**Authors:** Wolfram Weschenfelder, Katharina Lucia Koeglmeier, Friederike Weschenfelder, Christian Spiegel, Amer Malouhi, Nikolaus Gassler, Gunther Olaf Hofmann

**Affiliations:** 1 Department of Trauma, Hand and Reconstructive Surgery, University Hospital Jena, 07747 Jena, Germany; katharina.lucia.koeglmeier@uni-jena.de (K.L.K.); christian.spiegel@med.uni-jena.de (C.S.); gunther.hofmann@med.uni-jena.de (G.O.H.); 2 Department of Obstetrics, University Hospital Jena, 07747 Jena, Germany; friederike.weschenfelder@med.uni-jena.de; 3 Institute of Diagnostic and Interventional Radiology, University Hospital Jena, 07747 Jena, Germany; amer.malouhi2@med.uni-jena.de; 4 Institute of Forensic Medicine, Pathology Section, University Hospital Jena, 07747 Jena, Germany; nikolaus.gassler@med.uni-jena.de

**Keywords:** soft tissue tumour, lipoma, atypical lipomatous tumour, MRI, MDM2 amplification

## Abstract

**Background/Objectives**: This study aimed to develop a reliable scoring system combining clinical and radiological parameters to distinguish atypical lipomatous tumours (ALTs) from lipomas, improving diagnostic accuracy and reducing expensive molecular pathology testing. **Methods**: A retrospective analysis of 188 patients who underwent surgery for lipomatous tumours was conducted. Patient data, including medical history, pathology, and MRI imaging results, were reviewed. Four predictive models were developed using various clinical and imaging parameters, including age, tumour size, location, and MRI characteristics (homogeneity, contrast enhancement). Statistical analysis, including ROC curve analysis and logistic regression, was performed to assess the accuracy of these models. **Results**: The highest predictive accuracy was achieved with Model 1, which included seven parameters, yielding an AUC of 0.952. This model achieved a sensitivity of 96.4% and a negative predictive value (NPV) of 97.2%. Reducing the number of parameters lowered the accuracy, with contrast enhancement playing a significant role in Model 1. A risk calculator based on the optimal model was developed, offering an effective tool for clinical use that can be provided. Notably, 21 out of 37 ALTs lacked atypia and would have been missed without molecular testing. **Conclusions**: The developed scoring system, based on clinical and imaging parameters, accurately distinguishes ALTs from lipomas, offering a practical alternative to molecular pathology testing. This multi-parameter approach significantly improves diagnostic reliability, reducing the risk of misclassification and false negatives, while also potentially lowering healthcare costs.

## 1. Introduction

Lipomatous tumours represent the largest group of soft tissue tumours, accounting for approximately half of all benign soft tissue tumours and 25% of soft tissue sarcomas. Between benign lipomas and malignant liposarcomas, the new WHO classification categorizes atypical lipomatous tumours (ALTs) as intermediate grade [1]. Distinguishing lipomas from ALTs is challenging, as they share clinical features. While lipomas often require treatment for symptom or cosmetic reasons, ALTs necessitate excision due to risks of dedifferentiation and recurrence [2,3]. Accurate diagnosis is crucial for appropriate therapy and patient outcomes [4].

A multidisciplinary approach, including clinical, radiological, and pathological assessments, is essential for diagnosing adipose tumours. MRI and CT can differentiate benign from malignant tumours, with MRI being particularly useful [5]. Histopathological examination of biopsy samples further refines the diagnosis, with Murine Double Minute 2 (MDM2) amplification testing confirming ALT cases when needed. Fluorescence in situ hybridization (FISH) remains the gold standard due to its reliability, though it is resource intensive [6,7].

Alternative diagnostic approaches are currently being explored to optimize resource use [8,9]. Radiomics, AI-based imaging analysis, enhances tumour classification but requires further validation [4,10]. Nagano et al. proposed an MRI-based “ALT-Score” to differentiate ALTs from lipomas, but this is not yet used in routine radiological practice [11]. Contrast Enhanced Ultra-Sound (CEUS), a cost-effective alternative, has shown potential but lacks accuracy [12].

Molecular techniques like silver-enhanced in situ hybridization (SISH) and chromogenic in situ hybridization (CISH) offer cost-effective alternatives to FISH, but have lower sensitivity [13,14]. NanoString technology provides rapid, accurate results but remains limited in availability [15]. MDM2-FISH is recommended for high-risk cases, such as large or recurrent tumours in deep locations or older patients [8].

The primary objective of this study was to establish a scoring system consisting of clinical and radiological parameters to reliably distinguish between ALTs and lipomas.

## 2. Materials and Methods

### 2.1. Study Population

We retrospectively reviewed the medical records of patients who underwent surgery for lipomatous tumours at our university hospital between January 2005 and December 2024. Out of 246 eligible patients, 27 were excluded due to missing preoperative MRI imaging data or inadequate pathology reports. A further 31 patients with myxoid, pleomorphic, or dedifferentiated liposarcomas were excluded. A total of 188 patients were included in the final analysis. This study was approved by the relevant ethics committees of the authors’ affiliated institutions on 18 February 2025 (2025/3695-BO-D).

### 2.2. Data Collection and Prediction Models

Patient characteristics, medical history, pathology, and imaging results were collected from patient records. MRI imaging was re-evaluated regarding homogeneity and any, even marginal, contrast enhancement. To develop prediction models to distinguish lipomas, the above-mentioned parameters available at the time of presentation were tested in four different models.

Model 1 (“All Combined”) includes 7 discriminative preoperatively available variables that showed significant differences in the descriptive group comparison between patients with lipomas and ALTs: age (years), maximal diameter on MRI (centimetres), location in the lower limb, subfascial location proximal location in the extremity, homogeneity on MRI, and contrast enhancement on MRI.

Model 2 (“without contrast”) includes 6 of these 7 variables that were available if MRI was performed without contrast enhancement.

Model 3 (“including trunk location”) includes 6 of these 7 variables that were available if lesions of the soft tissues of the trunk were included.

Model 4 (“including trunk location without contrast”) includes 5 of these 7 variables that were available if MRI was performed without contrast enhancement and lesions of the soft tissues of the trunk were included.

### 2.3. Statistical Analysis

Statistical analysis was conducted using SPSS 29.0 (IBM©, New York, NY, USA). All patients meeting the inclusion criteria and not subject to any exclusion criteria were included in the analysis. The categorial data were compared with the results of Chi^2^ tests. The continuous data showed no normal distribution, so non-parametric tests had to be used to determine differences: the Mann–Whitney-U-test in the case of two categories; the Kruskal–Wallis-test in the case of more than two categories. The respective median with the inter quartile range is shown in the text.

For statistically significant metric variables, a cut-off was defined using receiver operating characteristics (ROC) curve analysis, with the application of the Youden index [16] based on the area under the curve (AUC) with a 95% confidence interval. The nominal parameters were also converted into binary variables accordingly.

A binary logistic regression model was then created with the binary versions of the significant clinical and imaging variables. The predictors of each respective prediction model *x*_1_, …, *x_k_* (where *k* represents the number of factors) were independent variables. The probability of a highly differentiated lipomatous tumour being an ALT was calculated using the following formula:

$$P(y=1)=\frac{1}{1+e^{-\left(\beta_{0}+\beta_{1}x_{1}+\beta_{2}x_{2}+\beta_{3}x_{3}+\ldots +\beta_{k}x_{k}+\varepsilon \right)}}$$

The parameters $\beta_{0}$, …, $\beta_{k}$ were the estimators for the coefficients of the multiple binary logistic regression model. A ROC analysis was performed to discriminate the two groups using the patient-specific probability of an ALT determined in the model. The cut-off value for distinguishing the groups was determined by the maximum Youden index and alternatively by defining the sensitivity at 1 to prevent false negative results. The negative and positive predictive values (NPVs and PPVs) were calculated for the various prediction models. A *p*-value of <0.05 was considered statistically significant.

## 3. Results

### 3.1. Baseline Characteristics

The study population consisted of 72 females and 116 males, with ages ranging from 18 to 90 years and a median age of 59 years (IQR: 47–69 years). Among them, 69 patients had lipomas with negative MDM2 amplification, 37 had proven ALTs, and 82 had a pathology report labelling them as lipomas but without molecular pathology testing. The parameters and models were created excluding the 82 patients without molecular pathology. Retrospective MDM2 status determination was not possible, as surgeries occurred before 2017 and specimens were no longer available. The baseline characteristics of these two groups are provided in Table 1.

### 3.2. Univariate Analysis of Relevant Parameters Distinguishing Lipomas and ALTs

**Figure 1:**
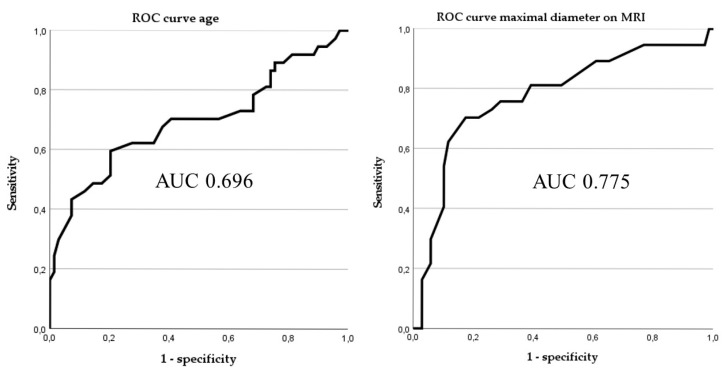
Receiver operating characteristics curves of age and maximal diameter on MRI.

The median age of patients presenting with an ALT was higher at 69 years compared to 60 years for those with a lipoma (*p* < 0.01). ROC curve analysis showed the best Youden index, with a cut-off of <68 years (AUC: 0.696; CI: 0.581–0.811; Figure 1).

Regarding tumour location, deep, lower limb, and proximal limb locations were significantly more common in patients with an ALT (*p* = 0.03, *p* < 0.01, and *p* < 0.01, respectively). Inhomogeneity on MRI and contrast enhancement were also significantly more frequent in ALTs (both *p* < 0.01).

Larger lesion size, measured both on MRI and in pathological specimens, was significantly associated with ALTs (both *p* < 0.01). Since MRI-based size measurement was available preoperatively, it was used in the further model. ROC curve analysis showed the best Youden index, with a cut-off of <15.5 cm (AUC: 0.775; CI: 0.675–0.875; Figure 1).

There was no significant difference between the two groups regarding gender, history of lesion growth, or time between first symptoms and presentation at our hospital.

As expected, ALTs exhibited significantly more atypia than lipomas (*p* < 0.01). However, 21 ALTs showed no atypia and would have been missed without molecular pathological examination. As per the definition, MDM2 amplification was significantly more common in ALTs (*p* < 0.01), although one ALT was negative, requiring additional cyclin-dependent kinase 4 (CDK4) testing to differentiate it from a lipoma in this specific case.

### 3.3. Predictive Accuracy of Different Prediction Tools

By combining significant clinical and imaging parameters in four models, a high AUC—and thus strong prediction accuracy—was achieved for distinguishing ALTs from lipomas. Model 1 (“all combined”), which included all seven parameters, yielded the highest AUC (0.952; CI: 0.910–0.995). The cut-off with the highest Youden index (J = 0.712) achieved a sensitivity of 0.964, a specificity of 0.833, a PPV of 0.794, and an NPV of 0.972.

Since it is clinically crucial not to miss any ALTs and to maximize the NPV, a second cut-off was defined, achieving an NPV of 1 and a sensitivity of 1. However, this adjustment resulted in a specificity of 0.762 and a PPV of 0.737.

**Figure 2:**
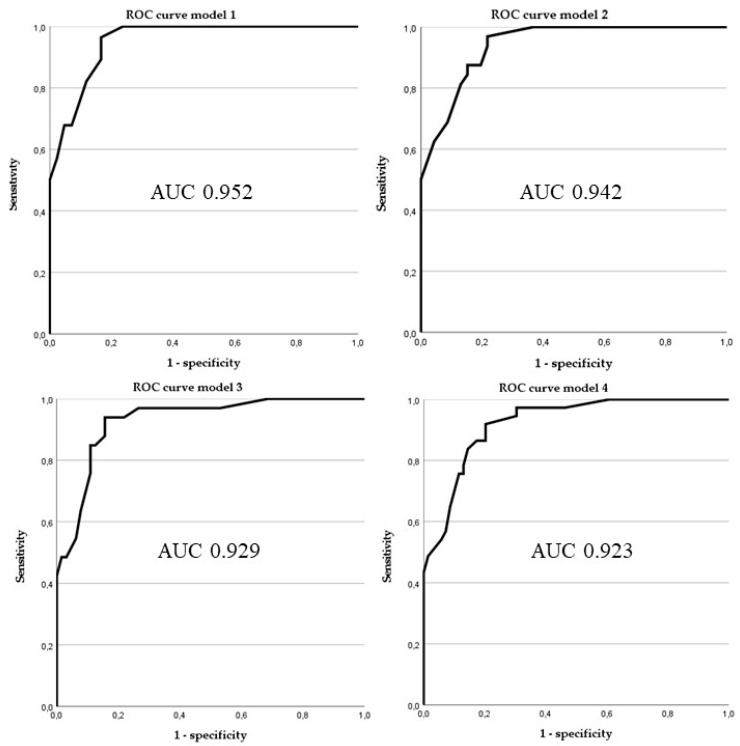
Receiver operating characteristics curves of models 1 to 4. Model 1: age over 67 years, size above 15.5 cm, lower limb, deep, proximal, homogeneity on MRI, and contrast enhancement on MRI. Model 2: age over 67 years, size above 15.5 cm, lower limb, deep, proximal, and homogeneity on MRI. Model 3: age over 67 years, size above 15.5 cm, lower limb, deep, homogeneity on MRI, and contrast enhancement on MRI. Model 4: age over 67 years, size above 15.5 cm, lower limb, deep, and homogeneity on MRI.

For Model 2 (“without contrast”), the AUC was 0.942 (CI: 0.896–0.987). In Model 3 (“including trunk location”), the AUC was 0.929 (CI: 0.878–0.981), and in Model 4 (“including trunk location without contrast”), the AUC was 0.923 (CI: 0.873–0.972). Sensitivity and NPV decreased as fewer parameters were included, leading to more false positive results when the cut-off was adjusted to favour a higher NPV (see Figure 2).

The predictive accuracy of histological atypia is lower than that of all models, particularly in terms of sensitivity. By definition, MDM2 amplification exhibits a very high predictive value in distinguishing ALTs from lipomas (see Table 2).

### 3.4. Assessment of Remaining 82 “Lipomas” Without Molecular Pathology Testing

Model 4 was used to assess these 82 patients, because contrast enhancement was not used in some of the MRIs, and tumours were also located in the trunk. According to the model, 14 cases had ALTs, which, from today’s perspective, were incorrectly assessed as lipomas.

### 3.5. Risk Calculator

The risk calculator will be made available online at the end of the publication process. Depending on the available data, the optimal model is automatically selected and the probability that a lipomatous tumour is an ALT is given as a percentage and with the cut-off.

## 4. Discussion

Based on the available data, we developed a prediction model with significantly higher accuracy than individual parameters or atypia as a postoperative marker. This risk calculator requires only four clinical and three simple imaging parameters to assess the likelihood that a lipomatous tumour is an ALT, thereby determining whether molecular pathology testing is necessary for the biopsy or resection specimen.

Consistent with previous studies, our database confirms that clinical factors such as tumour size, patient age, and deep or proximal localization in the lower extremity significantly differentiate ALTs from lipomas [10,17,18,19]. Additionally, a simple MRI evaluation distinguishing between homogeneous vs. non-homogeneous lesions and absence vs. presence of contrast enhancement also showed significant differences.

Combining all seven parameters resulted in an AUC of 0.952, yielding a very high NPV of 0.972 when using a cut-off based on the Youden Index, or an NPV of 1 when prioritizing maximum sensitivity. The combination of high NPV and relatively high specificity suggests that molecular pathology testing could be omitted in many cases, potentially reducing healthcare costs and resource use.

Our risk calculator’s predictive accuracy is nearly as high as the machine learning model developed by Cat et al. (AUC 0.987) and the clinical scoring system developed by Cheng et al. [19,20]. Unlike these models, our imaging evaluation is significantly simplified, making it more practical for routine use, as radiology reports already include homogeneity and contrast enhancement assessments.

By avoiding more complex imaging assessments—such as septation, percentage-based contrast enhancement, or inhomogeneity metrics—our model maintains high accuracy while minimizing interrater variability [19]. Imaging-only models tend to have lower accuracy and require additional image re-evaluation, consuming extra time and resources in clinical practice [10,17,18,21].

Removing the contrast enhancement parameter slightly reduced the AUC from 0.952 to 0.942 (model 1 to model 2), indicating its impact on predictive power. Therefore, unless contraindicated, contrast administration is recommended for MRI evaluation of lipomatous tumours. Additionally, our model demonstrated lower predictive power for tumours on the trunk (model 3: AUC 0.929), suggesting that for larger lesions in this region, MDM2 amplification analysis should still be performed as standard practice.

Overall, our study found that 21 out of 37 ALTs lacked histological atypia and were only identified through molecular pathology. Consequently, a significant proportion of presumed lipomas without MDM2 amplification analysis were likely ALTs according to our model. This underscores the high false negative rate when relying solely on histological evaluation without integrating clinical parameters or molecular pathology testing.

Moreover, case reports describe lipomatous tumours containing areas with and without MDM2 amplification positivity. This suggests that evaluating lesions based on multiple parameters may reduce the risk of missing abnormal regions due to tissue sampling errors in transitional forms [22]. To minimize the impact of interobserver variability in the assessment of histopathological findings, evaluations of soft tissue tumours at our centre are consistently performed by consensus, following the dual-observer principle. Nevertheless, the risk of sampling errors during the macroscopic selection of histological sections remains.

### Strength and Limitations

The number of cases in the presented study is similar to that in previous studies. One strength is the evaluation of the imaging using simple parameters while still maintaining a high level of accuracy. In view of the frequency of highly differentiated lipomatous tumours in the population, the study population is relatively small.

Currently, the score lacks external validation. It was developed using a patient cohort from a single sarcoma centre, which may introduce bias related to diagnosis and treatment practices. The score should be validated in an external patient collective.

## 5. Conclusions

The scoring system presented, which incorporates four simple clinical and three imaging parameters, demonstrated high diagnostic accuracy in distinguishing atypical lipomatous tumours from lipomas, and it is easily applicable compared to others. Additionally, our findings highlight the considerable variability within both tumour groups and the high false negative rate when relying on a single parameter—particularly the histological evaluation of atypia. These results underscore the importance of a multi-parameter approach to improving diagnostic reliability and reducing the risk of misclassification. Future developments in AI and radiomics-based evaluation models hold the potential to further improve diagnostic accuracy.

## Figures and Tables

**Table 1:** Baseline characteristics excluding lipomas without MDM2 amplification performed (number presented for ordinal data; median with inter quartile ranges presented for metric data).

	Lipoma *n* = 69	Atypical Lipomatous Tumour (ALT) *n* = 37	*p*-Values
Gender, male	45	22	0.56
Gender, female	24	15	
Age, in years	60 (48–67)	69 (56–78)	**<0.01**
History of increasing size of lesion			0.15
- yes	47	22	
- no	13	2	
Time between first symptoms and presentation, in months	24 (6–72)	11 (6–33)	0.19
Location			**<0.01**
- upper limb	29	2	
- lower limb	17	30	
- pelvis/trunk	23	5	
Location in relation to fascia			**<0.01**
- superficial	25	2	
- deep	44	35	
Location within limb			**0.03**
- proximal	34	30	
- distal	12	2	
Homogeneity on T1 and STIR on MRI			**<0.01**
- yes	45	11	
- no	24	26	
Any contrast enhancement on MRI			**<0.01**
- yes	32	26	
- no	32	7	
Largest diameter on MRI, in cm	9 (7–14)	18 (12–22)	**<0.01**
Largest diameter of pathological specimen, in cm	10 (7–12)	15 (10–21)	**<0.01**
Histologically proven atypia			**<0.01**
- yes	2	16	
- no	67	21	
Result of MDM2 amplification			**<0.01**
- positive	0	30	
- negative	69	1	

**Table 2:** Predictive accuracy of prediction models, histological atypia, and MDM2 amplification (models 1a, 2, 3 and 4, with cut-off calculated by Youden index; model 1b, with cut-off defined by negative predictive value of 1; * number of cases per total cases). Model 1: age over 67 years, size above 15.5 cm, lower limb, deep, proximal, homogeneity on MRI, and contrast enhancement on MRI. Model 2: age over 67 years, size above 15.5 cm, lower limb, deep, proximal, and homogeneity on MRI. Model 3: age over 67 years, size above 15.5 cm, lower limb, deep, homogeneity on MRI, and contrast enhancement on MRI. Model 4: age over 67 years, size above 15.5 cm, lower limb, deep, and homogeneity on MRI.

Model	Sensitivity	Specificity	Positive Predictive Value	Negative Predictive Value	Missed Atypical Lipomatous Tumours *	False Positive Lipomas *
Model 1a	0.964	0.833	0.794	0.972	1/28	7/42
Model 1b	1	0.762	0.737	1	0/28	10/42
Model 2	0.969	0.783	0.756	0.973	1/32	10/46
Model 3	0.939	0.844	0.756	0.964	2/33	10/64
Model 4	0.919	0.797	0.708	0.948	3/37	14/69
Histological atypia	0.432	0.971	0.889	0.761	21/37	2/69
MDM2 amplification	0.970	1	1	0.986	1/33	0/69

## Data Availability

The data presented in this study are not publicly available but available upon request from the corresponding author. The data are not publicly available due to privacy and ethical restrictions.

## Abbreviations

The following abbreviations are used in this manuscript:

AI	Artificial Intelligence
ALT	Atypical lipomatous tumour
AUC	Area under the curve
CDK4	Cyclin-dependent kinase 4
CEUS	Contrast Enhanced Ultra-Sound
CISH	Chromogenic in situ hybridization
CT	Computed Tomography
FISH	Fluorescence in situ hybridization
IQR	Inter quartile range
MDM2	Murine Double Minute 2
MRI	Magnetic Resonance Imaging
NPV	Negative Predictive Value
PPV	Positive Predictive Value
ROC	Receiver operating characteristics
SISH	Silver-enhanced in situ hybridization
WHO	World Health Organization
